# Unilateral endonasal kaposiform haemangioendothelioma in a newborn – a case report

**DOI:** 10.1007/s00405-026-10231-x

**Published:** 2026-04-23

**Authors:** Dominik Lanzerstorfer, Johannes Hochstöger, Mario Scala, Nikolaus Poier-Fabian, Paul Zwittag

**Affiliations:** 1 Department of Otorhinolaryngology, Head and Neck Surgery, Kepler University Hospital GmbH, Krankenhausstrasse 9, 4021 Linz, Austria; 2 Department of Radiology, Kepler University Hospital GmbH, Krankenhausstrasse 9, 4021 Linz, Austria; 3 Medical Faculty, Johannes Kepler University Linz, Altenberger Strasse 69, 4040 Linz, Austria

**Keywords:** Kaposiform Haemangioendothelioma, Kasabach-Merritt phenomenon, Newborn, Sinonasal Tumour

## Abstract

**Introduction:**

Kaposiform haemangioendothelioma (KHE) is a rare, borderline, vascular tumour occurring mostly during childhood. Literature provides reports of KHE in various body regions. To the best of our knowledge, this is the first report of an endonasal KHE. Expert panels recommend systemic pharmacotherapy as first line treatment. In this case, physicians opted for surgical excision of the tumour instead. This report presents therapeutic challenges in this unique case and the succesful course of treatment.

**Main symptoms/clinical findings:**

The newborn patient presented with saturation drops below 80% SpO_2_ while breastfeeding, and recurrent episodes of bloody nasal secretions. Endoscopy showed a large, obstructing and highly vascularized tumour in the right nasal cavity. In imagery, the tumour extended from the olfactory fossa to the nasal floor and eroded the bony border of the medial orbit.

**Main diagnoses/therapeutic interventions/outcomes:**

In summary of the findings, physicians assumed teratoma or glioma as working diagnosis. During surgery, extensive haemorrhage required the transfusion of approximately 50% of the patient`s total blood volume. Following a satisfactory recovery, the patient was discharged five days after surgery. The patient presented well and tumour free in all follow-up examinations. Histopathological examination confirmed Kaposiform haemangioendothelioma as tumour entity.

**Conclusion:**

KHE in the upper airway can pose serious life-threats to newborns. Risks associated with diagnostic examination should be weighed up against the accepted level of uncertainty in treatment process decisions. Including caregivers in treatment decisions is recommendable. Despite the absence of KMP, haemorrhage can require transfusion of erythrocyte concentrates. Managing extensive haemorrhage requires preparation, equipment and skill.

## Introduction

Kaposiform haemangioendothelioma (KHE) is a rare, borderline vascular tumour [1] with a prevalence of 0.91 per 100,000 children [2]. KHE usually presents during infancy or early childhood [3] and shows a tendency of local invasion and recurrence [4]. Usually, KHE is discovered in the retroperitoneum, on the trunk or the extremities [5] as well as in the head and neck area [6]. To the best of our knowledge, this is the first report of an endonasal KHE. This case report aims at presenting the therapeutic challenges in this unique case and the succesful course of treatment (Table 1) to a broad audience in the medical community.

## Timeline

**Table 1:** Case report timeline

Referral to ENT Department	2024–10-01
MRI Scan of the Skull	2024–10-02
CT Scan of the Skull	2024–10-03
Surgery	2024–10–16
Discharge	2024–10–21
Follow-up Examination 1	2024–11–19
Follow-up Examination 2	2025–01–16
Follow-up MRI	2025–08–11
Follow-up Examination 3	2025–08–11

## Patient information and Diagnostic assessment

The maternity ward physician referred the two-day old patient to the department of otorhinolaryngology at Kepler University Hospital Linz because of saturation drops to below 80% spO2 while breastfeeding, bloody nasal secretions and tear duct congestion on the right side. In the clinical examination the patient displayed no other pathologies.

**Fig. 1:**
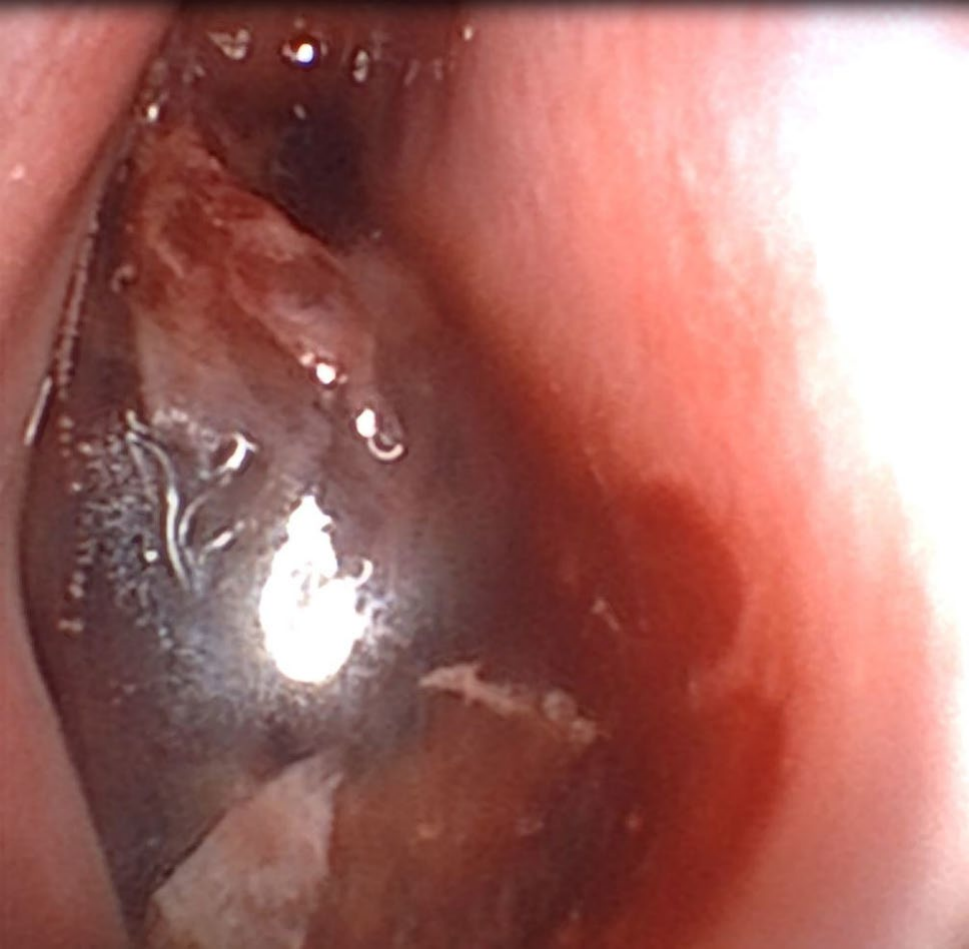
Kaposiform Haemangioendothelioma located in the right nasal cavity visualised in flexible transnasal endoscopy

**Fig. 2:**
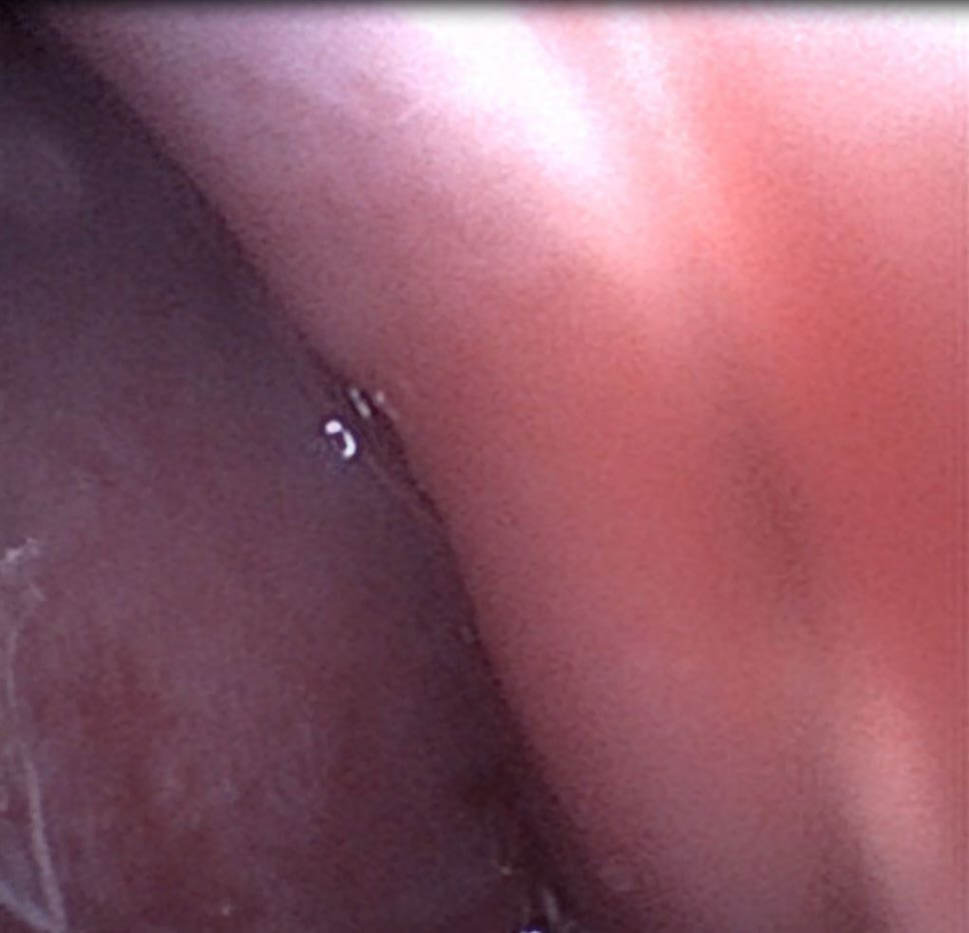
Nasal septum displacement to the left visualised in flexible transnasal endoscopy

**Fig. 3:**
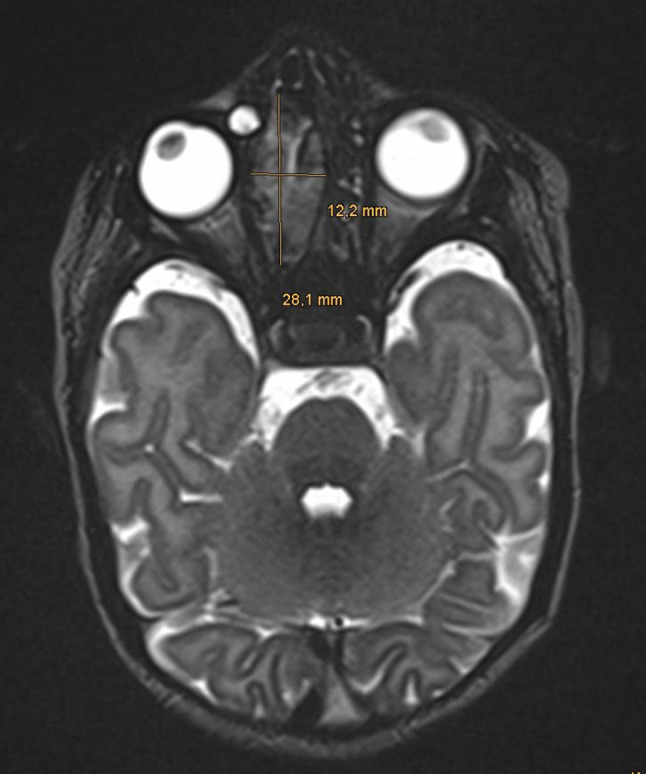
Expansion of Kaposiform Haemangioendothelioma in axial plane in T2 weighted MRI scan, note the compression of the right tear duct

**Fig. 4:**
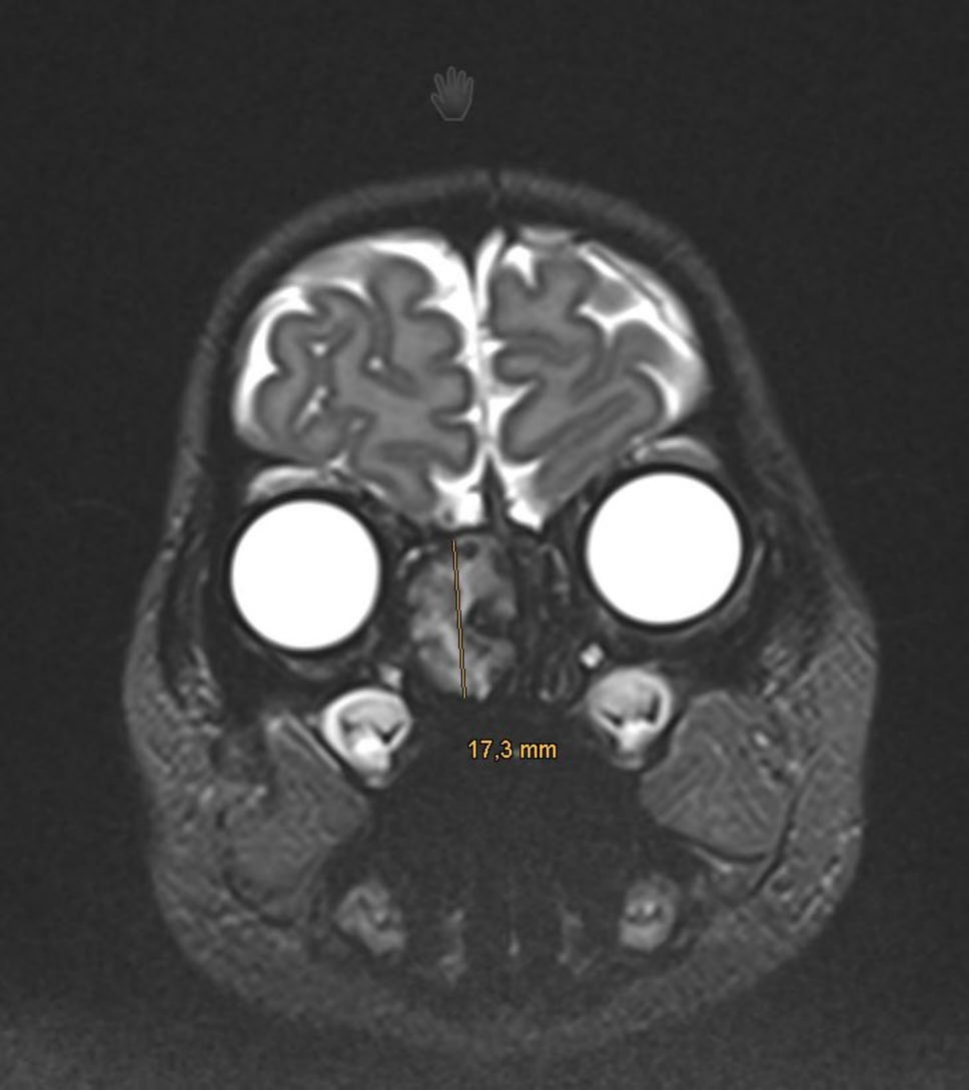
Expansion of Kaposiform Haemangioendothelioma in T2 weighted MRI scan in coronal plane

**Fig. 5:**
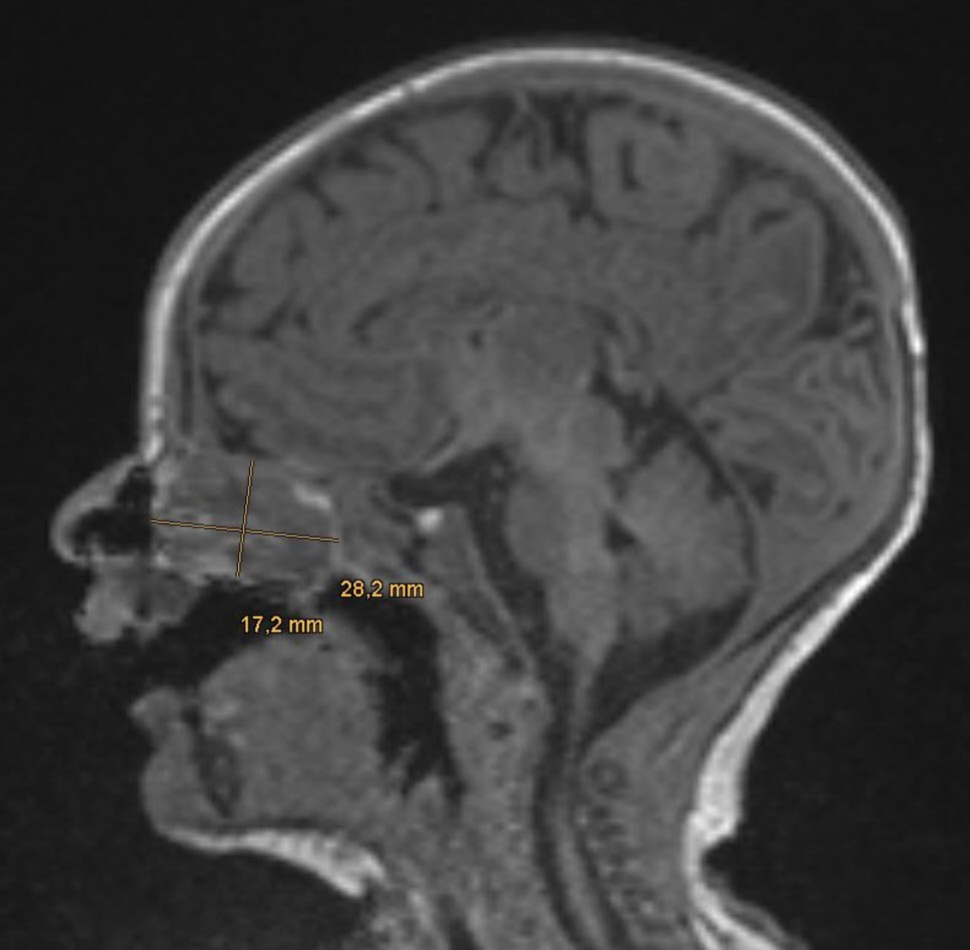
Expansion of Kaposiform Haemangioendothelioma in T1 weighted MRI scan in sagittal plane

**Fig. 6:**
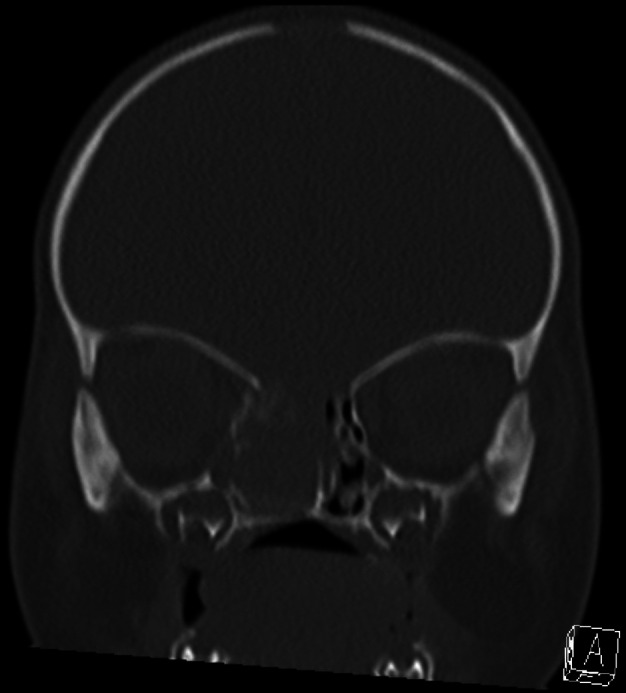
Invasion of the bony border of the orbit and scarification of the frontal skull base in CT scan in coronal plane

Nasal endoscopy showed a highly vascularized tumour in the right nasal cavity (Fig. 1) displacing the nasal septum to the left side (Fig. 2) and obstructing the airway. In magnetic resonance and computer tomography images the tumour extended from the nasal vestibule to the choana and from the nasal floor up to the skull base. The tumour measured 29 mm in the sagittal plane, 15 mm in the axial plane (Fig. 3), and 12 mm in the coronal plane (Fig. 4). Also, it extended into the olfactory fossa (Fig. 5) and eroded the bony border of the medial orbit (Fig. 6). The tumour mass showed calcification in its laterosuperior proportion. Furthermore, the tumour compressed the right inferior turbinate, and congested the right tear duct (Fig. 3). The periorbita and the neurocranium showed no signs of tumour invasion. The dura mater presented intact. Preoperative laboratory evaluation presented adequate results.

The KHE diagnosis requires clinical, histological and imaging features [1]. Due to the haemorrhage risk and the airway obstruction physicians omitted the diagnostic biopsy and relied on the clinical pathology and the imagery results that argued for KHE. Radiologists assumed teratoma or glioma as likely tumour entities due to the visible calcification in the tumour mass.

Multidisciplinary expert panels recommend systemic pharmacotherapy regimens as first line treatment [5]. In accordance with patient caregivers physicians opted for surgical excision and scheduled surgery fifteen days after patient referral. The time delay was accepted to reduce the risks associated with general anaesthesia in newborns. Erythrocyte concentrates, fresh frozen plasma, and fibrinogen were prepared for the surgery (Table 2).

**Table 2:** Laboratory evaluation results

	preoperative	intraoperative	postoperative
platelet count (G/L)	421	347	291
fibrinogen (mg/dL)	214	177	154 (decreased)
partial thromboplastin time (sec.)	34.3 (prolonged)	52.4 (prolonged)	33.8 (prolonged)
Prothrombin time (%)	74 (shortened)	46 (shortened)	64 (shortened)

## Therapeutic intervention

As expected, extensive haemorrhage occurred right after the initial frozen section biopsy to confirm the histopathological tumour entity. Repeated transient packing with epinephrine-soaked cotton pledges and cautious use of the monopolar suction catheter provisionally stopped the haemorrhage. Since macroscopic examination did not confirm the suspected bony invasion of the skull base and intraoperative biopsy results remained inconclusive the tumour was removed en-bloc without wide resection distance to prevent cerebrospinal fluid leakage. After tumour excision, skull base sites were coagulated with a bipolar current instrument. To ensure adequate wound closure and seal potential unnoticed liquor fistula, the operation field was covered with Tachosil bricks, fibrin glue, and resolvable sinonasal packing. Continuous blood loss during surgery required the transfusion of 130 ml erythrocyte concentrate equalling approximately 50% of the patient`s total blood volume. Furthermore, the patient received 100 ml of fresh frozen plasma and 2 g of fibrinogen during surgery.

After surgery, the inferior nasal meatus remained open for respiration and irrigation. Resected tumour material was sent to the department of pathology at University Clinic Linz, the department of pathology at the Medical University Vienna and the institute of pathology at the University Hospital Bonn. Overall, the surgical procedure lasted approximately three hours.

## Follow-up and outcomes

Following a satisfactory recovery the patient was discharged five days after surgery without signs of bleeding or liquor fistula. Tear duct obstruction completely resolved shortly after surgery.

**Fig. 7:**
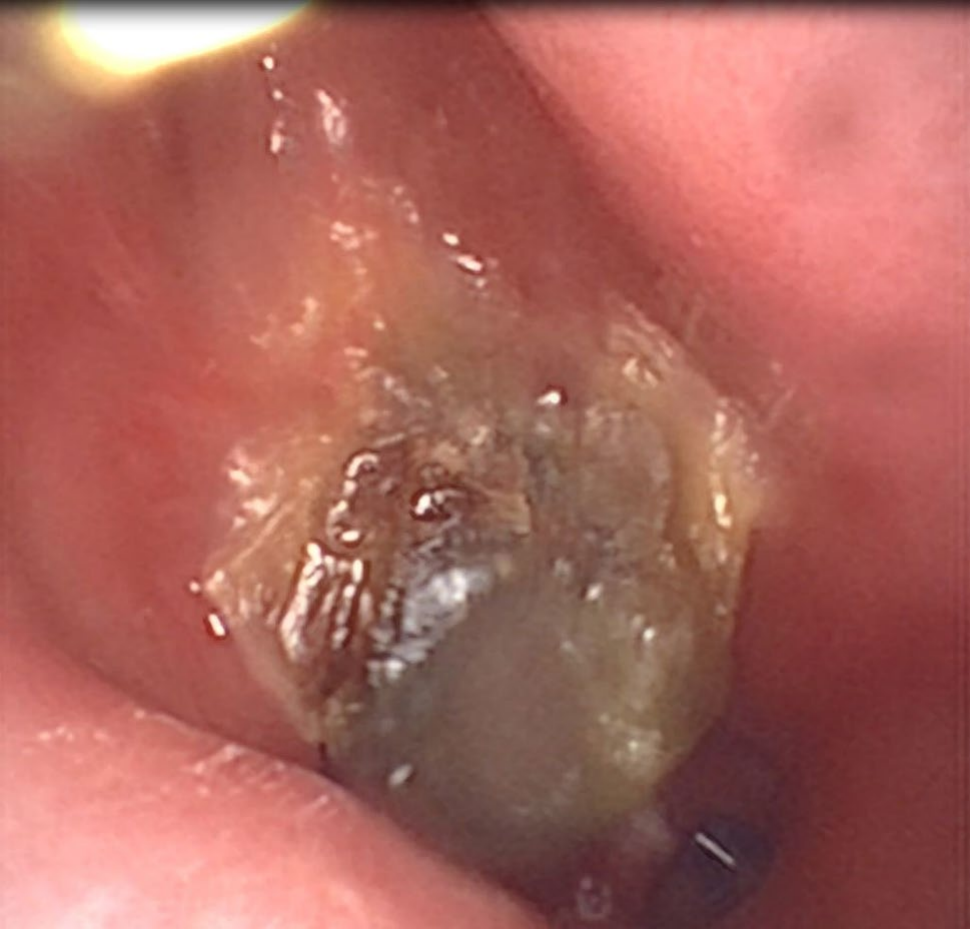
Right anterior skull base one month after surgery displaying crusts on the former tumour origin site visualised in flexible transnasal endoscopy

**Fig. 8:**
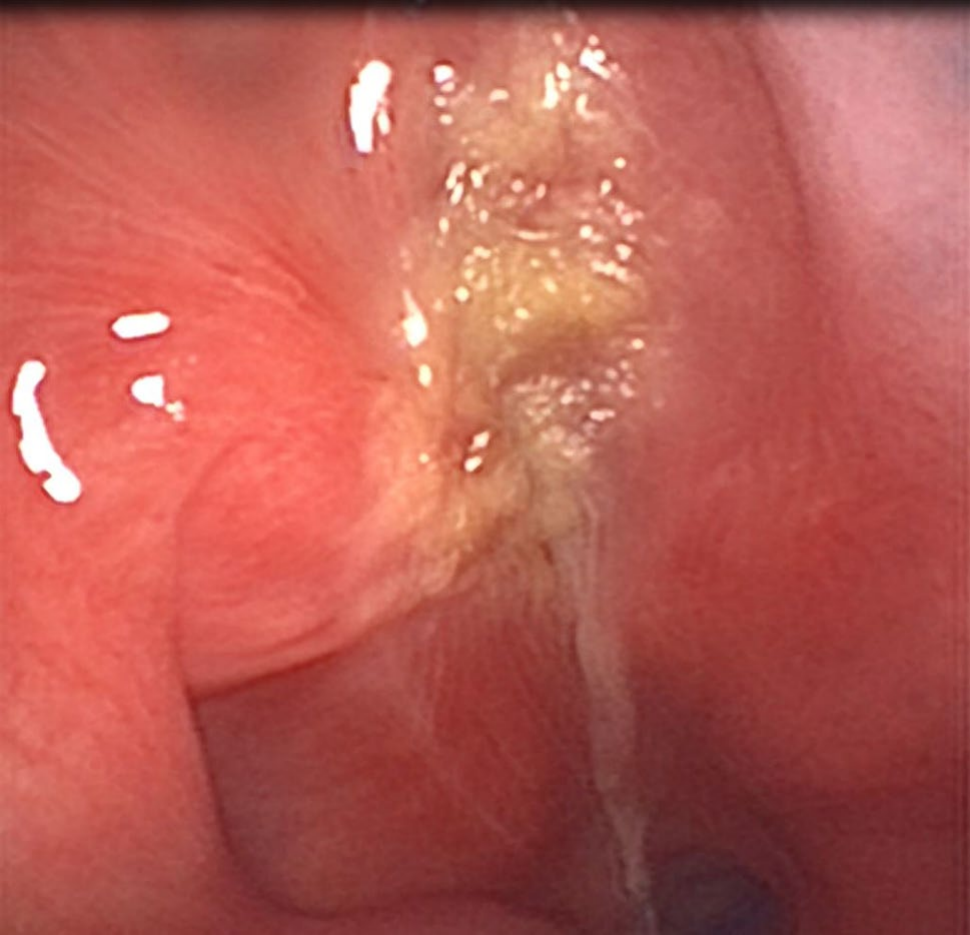
Former tumour origin site three months after surgery visualised in flexible transnasal endoscopy

**Fig. 9:**
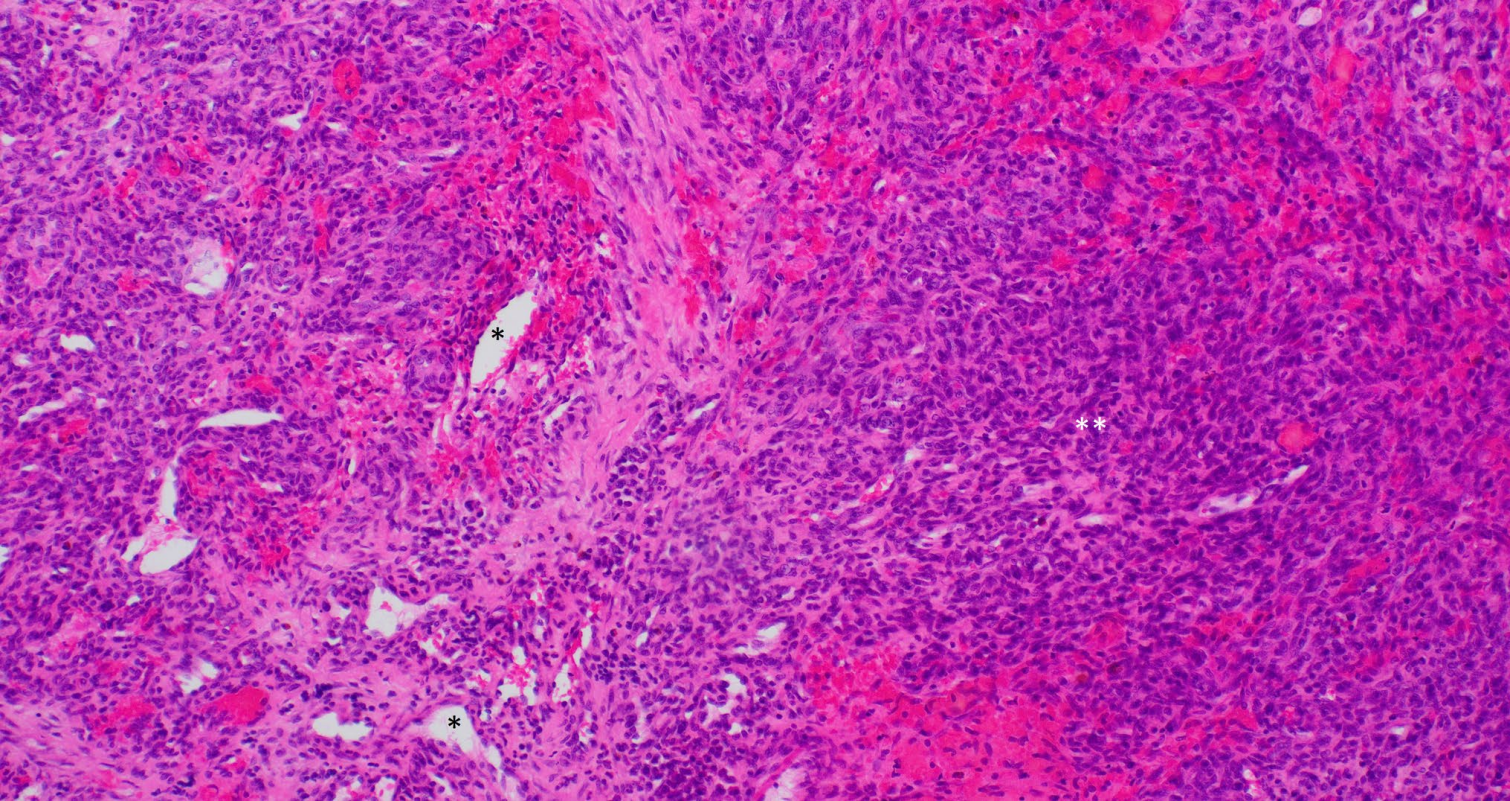
Histopathological photomicrograph of the kaposiform haemangioendothelioma after haematoxylin and eosin staining 200x—* pre-exisiting blood vessels; ** tumour

**Fig. 10:**
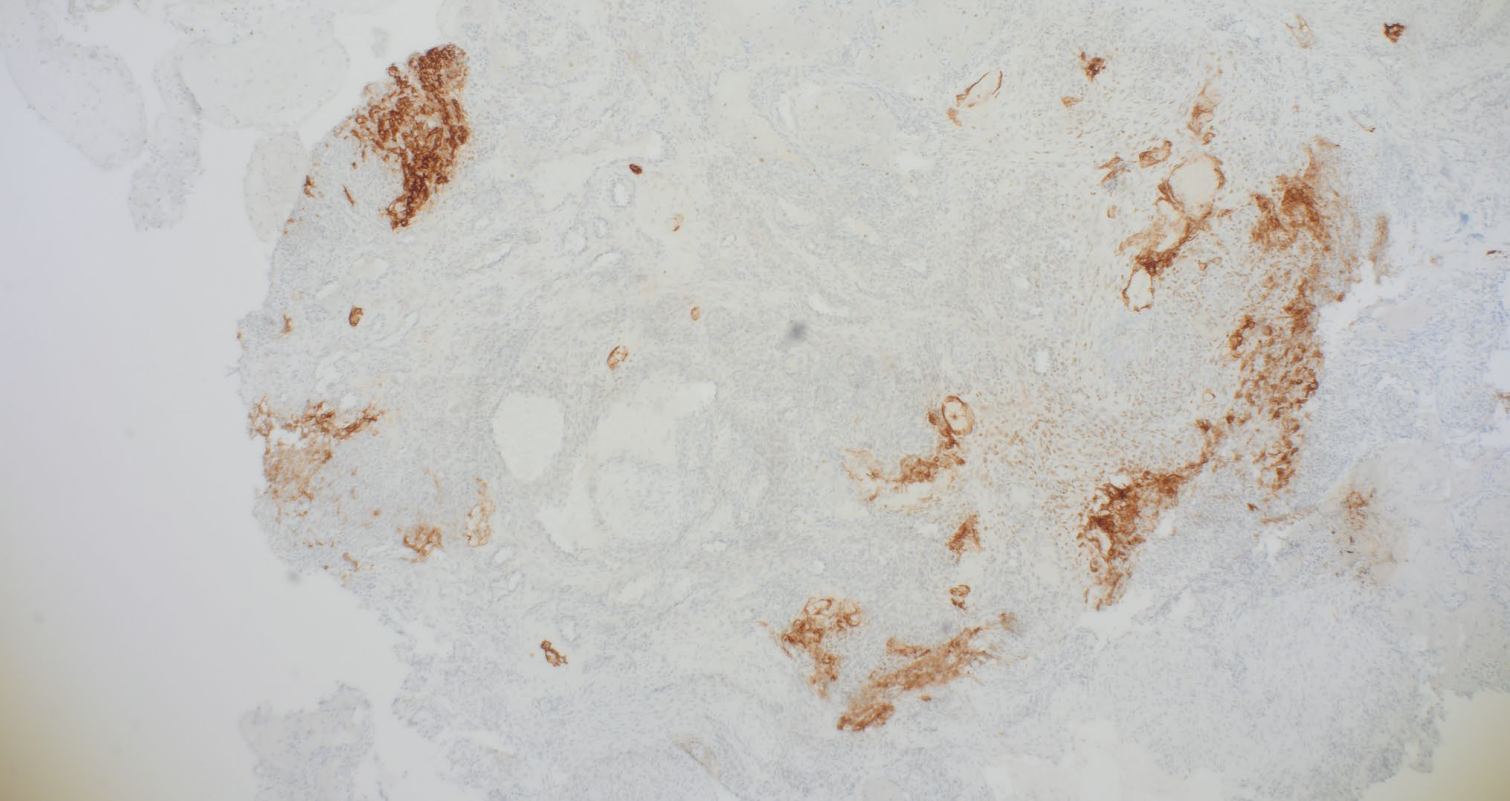
Histopathological overview photomicrograph of kaposiform haemangioendothelioma with podoplanin expression in the immunohistochemistry (brown staining) 40x

Nasal endoscopy in the follow-up examinations one month (Fig. 7) and three months after surgery (Fig. 8) showed inconspicuous postoperative results. The examination of the tumour material by the department of pathology at the Medical University Vienna resulted in the diagnosis of kaposiform haemangioendothelioma (Fig. 9), while the examination of the institute of pathology at the University Hospital Bonn resulted in the diagnosis of congenital haemangioma. Finally, after intra- and interdisciplinary discussion, histopathological bone invasive growth of the tumour, the podoplanin expression (Fig. 10) and the clinical course led to the diagnosis of kaposiform haemangioendothelioma.

**Fig. 11:**
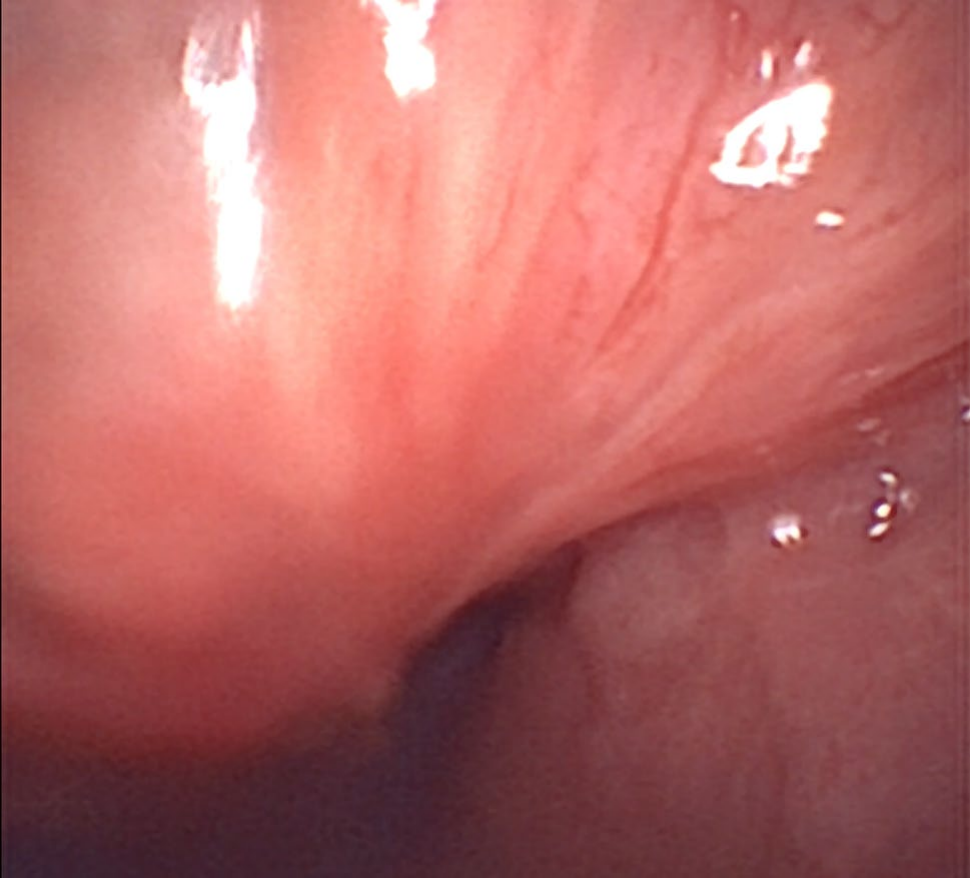
Former tumour origin site ten months after surgery visualised in flexible transnasal endoscopy

**Fig. 12:**
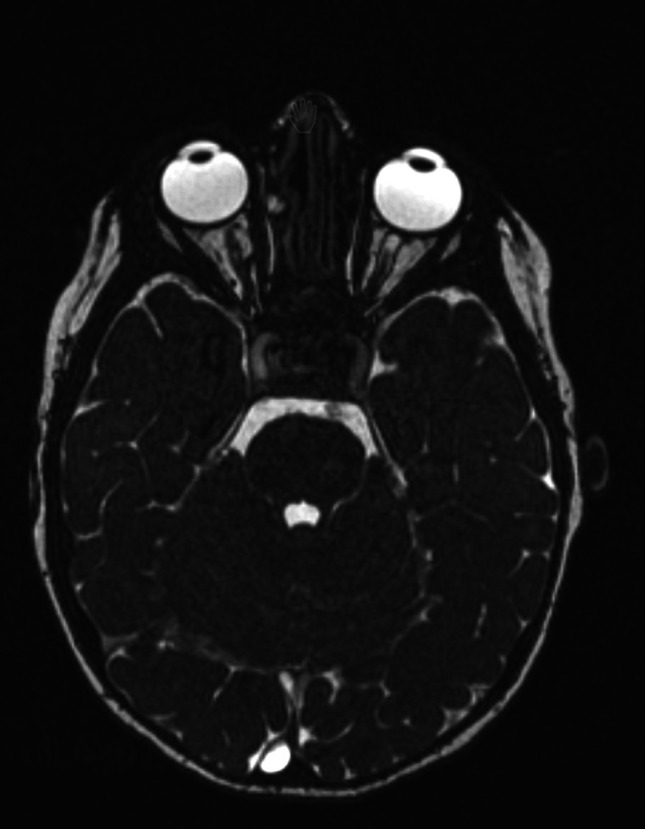
T2 weighted MRI scan in axial plane ten months after surgery

**Fig. 13:**
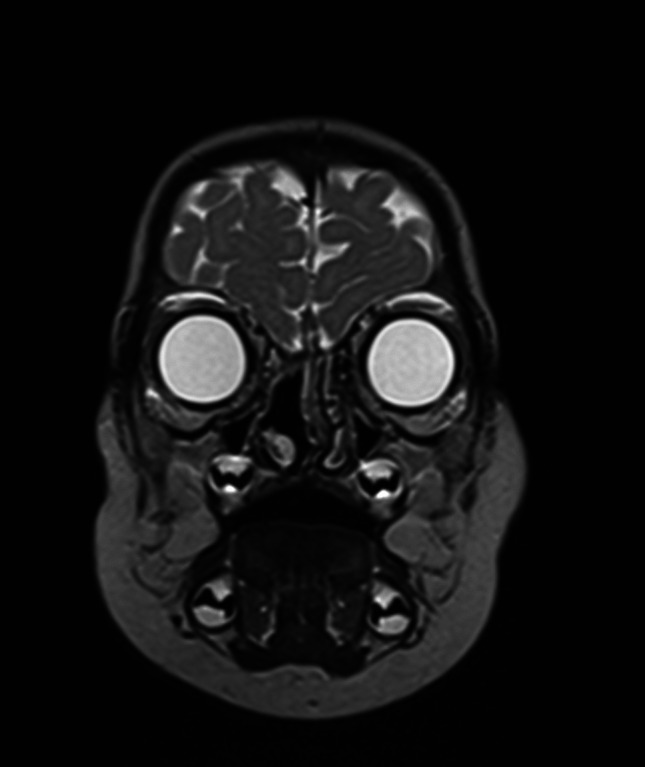
T2 weighted MRI scan in coronal plane ten months after surgery

**Fig. 14:**
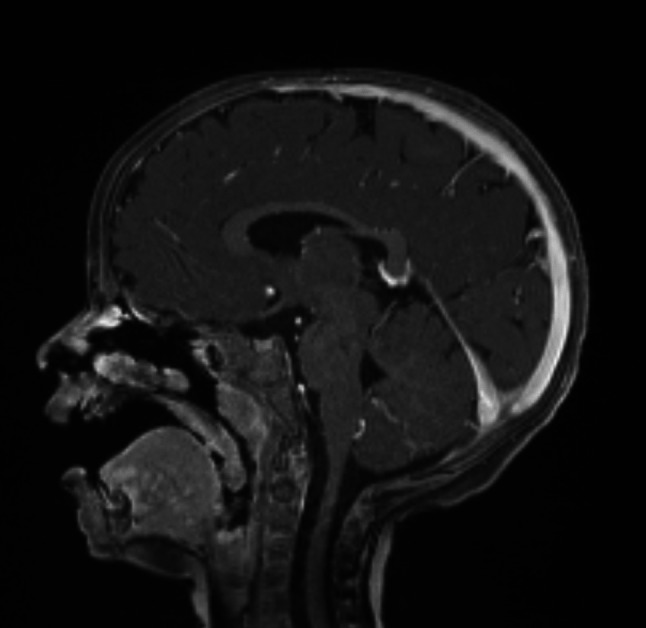
T2 weighted MRI scan in sagittal plane ten months after surgery

Nasal endoscopy ten months after surgery showed an inconspicuous postoperative result (Fig. 11). Furthermore, magnetic resonance images ten months after surgery presented a tumour free patient (Fig. 12, Fig. 13 and Fig. 14). Furthermore, the patient showed no postoperative complications such as dry nose symptoms, liquor fistulation and predisposition to epistaxis.

## Discussion

Despite its borderline tumour entity [1] kaposiform haemangioendothelioma in the upper airway can pose serious life-threats to a newborn due to airway obstruction and its tendency to invade bone tissue. Furthermore, the majority of KHE patients develops Kasabach-Merritt phenomenon (KMP), a consumptive coagulopathy that increases their haemorrhage risk [7].

According to literature, the KHE diagnosis requires clinical, histopathological and imaging features [1]. Due to the tumour`s airway obstruction the necessity of a diagnostic biopsy acquired before surgery was questioned. Therefore, physicians intentionally omitted the preoperative biopsy although it is the gold standard in histopathological KHE diagnosis [8]. This level of uncertainty regarding the tumour entity was accepted, due to the airway obstruction and the patient`s age. Relying on clinical and imaging features physicians opted for surgical excision in accordance with patient caregivers, although systemic pharmacotherapy is recommended as first line treatment [5]. Since KHE predominantly occurs during infancy and childhood [3], patient caregivers should be included in treatment decisions.

Despite the absence of Kasabach-Merritt phenomenon (Table 2), our patient suffered extensive haemorrhage during surgery that required the transfusion of erythrocyte concentrate equalling approximately 50% of the patient`s total blood volume. The extensive intraoperative haemorrhage was manageable in the operating theatre of a tertiary university clinic. However, it remains uncertain if this haemorrhage had been manageable in a less experienced or well-equipped setting. Transfusion readiness is essential in planning KHE excisions.

Although frozen section results remained inconclusive during surgery, the tumour was resected without wide resection distance to prevent postoperative leakage of cerebrospinal fluid. Due to KHE`s borderline entity, the patient required no further treatment. With a malign histopathological result the patient would have likely required post-resection or adjuvant radiation therapy to prevent recurrence and metastasis, depending on tumour board recommendations. An expert consensus statement published two months after surgery supported this treatment decision to surgically remove the tumour [1]. In retrospect, it remains questionable whether preoperative knowledge of the tumour entity would have changed the treatment decision.

Due to the succesful en-bloc tumour excision and the inconspicuous results of the follow-up examinations (Fig. 7, Fig. 8, Fig. 11, Fig. 12, Fig. 13 and Fig. 14) the patient`s life expectancy remains unaffected. Potential long-term complications such as dry nose symptoms, liquor fistulation and predisposition to epistaxis did not occur during the time span of the postoperative follow-ups. The reconstructed skull base perforation will likely require no further treatment. Since endoscopic transnasal procedures in newborns are rare events there is lack of suitable instruments. The instruments used in this procedure were borrowed from other otorhinolaryngology subspecialties.

## Conclusion

Kaposiform haemangioendothelioma in the upper airway can pose serious life-threats to a newborn. The haemorrhage risk associated with diagnostic biopsy outweighed the value of preoperative tumour entity knowledge for the treatment process decision. Although systemic pharmacotherapy is recommended as first-line treatment for KHE, physicians opted for early surgical excision because of the airway obstruction and the elevated haemorrhage risk. Despite extensive intraoperative haemorrhage, the tumour was reseceted en-bloc and the patient made a full recovery. The patient presented tumour free in all follow-up examinations and his life expectancy remains unaffected.

## Data Availability

Data are available from the corresponding author upon reasonable request.
